# S246. PSYCHOMETRIC PROPERTIES OF THE DANISH VERSION OF BNSS (BNSS-DA)

**DOI:** 10.1093/schbul/sby018.1033

**Published:** 2018-04-01

**Authors:** Johannes Gehr, Birte Glenthoj, Mette Nielsen

**Affiliations:** 1 Center for Neuropsychiatric Research, Copenhagen University Hospital; 2 Center for Neuropsychiatric Schizophrenia Research, CNSR, and Center for Clinical Intervention and Neuropsychiatric Schizophrenia Research, Mental Health Centre Glostrup, University of Copenhagen

## Abstract

**Background:**

The concept of negative symptoms (NS) has been known since early 19th century but the development of assessment instruments and treatment methods has yet proved inadequate. The Brief Negative Symptoms Scale (BNSS) was designed to evaluate NS according to a consensus definition by the National Institute of Mental Health from 2005. This study examines the validity and reliability of the Danish version of BNSS (BNSS-Da).

**Methods:**

49 participants with schizophrenia or schizoaffective disorder were included, counting in- and outpatients as well as users of community housing facilities. Participants were assessed with BNSS-Da, Positive And Negative Syndrome Scale (PANSS), Scale for the Assessment of Negative Symptoms (SANS), Calgary Depression Scale for Schizophrenia (CDSS), St. Hans Rating Scale for extrapyramidal syndromes (SHRS), Personal and Social Performance Scale (PSP), Trail Making Test A and B, (TMT-A/B) and Digit Symbol Substitution Test (DSST). 19 of included subjects had their BNSS-Da interviews rated separately by two raters in order to evaluate interrater agreement. The convergent and divergent validity of BNSS-Da was assessed by its relationship with the aforementioned scales and tests.

**Results:**

Of 49 included subjects, 45 were diagnosed with schizophrenia and 4 with schizoaffective disorder. The mean age was 33.1 (SD: 10.8) years and 65.3% were male. Mean duration of illness was 9.7 (SD: 9.2) years and the mean PANSS total score was 65.7 (SD: 17.6). Interrater reliability for BNSS-Da was estimated by calculating the intraclass correlation coefficient based on a mean-rating (k=2), absolute-agreement, 2-way mixed-effects model, which showed to be 0.953 (95%CI: 0.880–0.982). To examine convergent and divergent validity, Spearman’s rank correlation coefficients were calculated. PANSS negative and SANS total (subgroup, n=38) were both well correlated with BNSS-Da (ρ=0.813, p<0.001 and ρ=0.852, p<0.001). Also, BNSS-Da seemed to correlate well with PANSS total (ρ=0.736, p<0.001), and to a lesser extend PANSS positive (ρ=0.552, p<0.001) and PANSS general (ρ=0.628, p<0.001). More infirm correlations were found between BNSS-Da and CDSS (ρ=0.314, p=0.028), PSP (ρ=-0.480, p<0.001), DSST (subgroup, n=47, ρ=0.393, p=0.006) and TMT-B (subgroup, n=39, ρ=0.357, p=0.025), while no significant correlations were found with TMT-A (subgroup, n=39) or SHRS, except the Parkinsonism subscale (ρ=0.420, p=0.003).

**Discussion:**

The interrater reliability for the BNSS-Da proved to be excellent. Regarding convergent validity, the scale correlated well with the standardized assessment tools for NS, indicating the presence of a common construct of NS. Social functioning, as measured by PSP, was fairly correlated with BNSS-Da, demonstrating how NS are associated with functional outcome. As for divergent validity, the poor correlations between BNSS-Da and CDSS and most domains of SHRS suggested a good capacity for distinguishing between primary NS and NS secondary to depression or adverse effects of neuroleptics. However, the Parkinsonism subscale of SHRS had a rather firm correlation with BNSS-Da, probably because lack of facial expressions is measured in both scales. PANSS positive also seemed to correlate with BNSS-Da. This correlation assumedly stems from NS secondary to positive symptoms, since the present study included acutely psychotic subjects, unlike most other studies on translations of BNSS. The cognitive tests, TMT-A/B and DSST, were infirmly correlated to BNSS-Da, illustrating that cognitive function and NS likely are associated yet still separable through BNSS-Da. In conclusion, BNSS-Da holds appropriate psychometric properties in terms of reliability and validity.

